# The HBV DNA cutoff value for discriminating patients with HBeAgnegative chronic hepatitis B from inactive carriers

**Published:** 2011-05-01

**Authors:** Eun Sun Kim, Yeon Seok Seo, Bora Keum, Ji Hoon Kim, Hyonggin A, Hyung Joon Yim, Yong Sik Kim, Yoon Tae Jeen, Hong Sik Lee, Hoon Jai Chun, Soon Ho Um, Chang Duck Kim, Ho Sang Ryu

**Affiliations:** 1 Departments of Internal Medicine, Korea University College of Medicine, Seoul, Korea; 2 Biostatistics, Korea University College of Medicine, Seoul, Korea

**Keywords:** Hepatitis B virus, Reactivation, Inactive carrier

## Abstract

**Background:**

Patients with HBeAg-negative chronic hepatitis B (CHB) has a significantly different prognosis than inactive carriers; there is however, no reliable strategy for accurately differentiating these two disease conditions.

**Objectives:**

To determine a strategy for discriminating patients with HBeAg-negative CHB from inactive carriers.

**Materials and Methods:**

Consecutive inactive carriers (i.e. HBeAg-negativity, anti-HBe-positivity, normal ALT levels, and HBV DNA < 2000 IU/mL) were enrolled. HBV reactivation was defined as the elevation of the HBV DNA level to ≥ 2000 IU/mL. Patients were classified into true inactive carriers when their HBV DNA levels remained at < 2000 IU/mL or false inactive carriers when their HBV DNA levels increased to ≥ 2000 IU/mL during the first year.

**Results:**

The Mean ± SD age of 208 inactive carriers (140 males) was 47.7 ± 12.6 years. The Mean ± SD serum ALT and HBV DNA levels were 22.8 ± 8.6 IU/L and 360 ± 482 IU/mL, respectively. HBV reactivation developed in 41 (19.7%) patients during the first year. Baseline HBV DNA and ALT levels differed significantly between true inactive and false inactive carriers. The AUROCs of the baseline ALT and HBV DNA levels for predicting a false inactive carrier were 0.609 and 0.831, respectively. HBV reactivation developed more often in patients with a baseline HBV DNA level of ≥ 200 IU/mL than in those with a baseline HBV DNA level of < 200 IU/mL during a Mean ± SD follow-up of 622 ± 199 days.

**Conclusions:**

The HBV DNA level was useful for discriminating patients with HBeAg-negative CHB from true inactive carriers. The follow-up strategies applied to inactive carriers need to vary with their HBV DNA levels.

##  Background

The loss of HBeAg in hepatitis B virus (HBV) carriers was considered a sign of remission of hepatitis and suppression of HBV replication [1][2]. However, the application of sensitive methods for detecting HBV in the serum showed that HBV could continuously or intermittently replicate even in an HBeAg-negative patient [3][4]. HBeAg-negative HBV carriers are not a homogeneous group, with their clinical spectra ranging from inactive carrier to aggressive HBeAg-negative hepatitis or cirrhosis [5][6]. The inactive carrier state is characterized by HBeAg-negativity and anti-HBe-positivity, low or undetectable HBV DNA, normal alanine aminotransferase (ALT) levels, and inactive liver histology [7][8]. HBV carriers in this state usually have an excellent prognosis and rarely progress to cirrhosis or hepatocellular carcinoma [9][10]. Therefore, regular follow-up is recommended without antiviral therapy [11][12]. In contrast, antiviral treatment is recommended for patients with HBeAg-negative chronic hepatitis B (CHB), because sustained spontaneous remission is uncommon and their long-term prognosis is poor [13]. Therefore, discrimination of patients with HBeAg-negative CHB from inactive carriers is very important for the improving of prognosis in HBV carriers. However, this might be difficult in some cases. The HBV DNA level is usually lower than in patients with HBeAg-positive CHB than in those with HBeAg-negative CHB [14]. In addition, HBV DNA levels can fluctuate widely in patients with HBeAg-negative CHB [14][15], which can result in some patients with HBeAg-negative CHB being misclassified as inactive carriers.

Because recent studies have suggested that the risks of both cirrhosis and hepatocellular carcinoma are significantly higher in HBV carriers with an HBV DNA level of ≥ 2000 IU/mL [7][8][16][17], most of the recent guidelines recommend an HBV DNA cutoff level for inactive carriers of 2000 IU/mL [11][12]. However, since HBV DNA levels can fluctuate to even lower than 2000 IU/mL in patients with HBeAg-negative CHB, an HBV carrier should not be classified as being inactive solely based on a single HBV DNA reading of < 2000 IU/mL, which has led to appropriate follow-up being recommended for differentiating inactive carriers and patients with HBeAg-negative CHB [11][12]. However, the optimal follow-up strategy for this differentiation remains uncertain. The American Association for the Study of Liver Diseases (AASLD) practice guideline recommends inactive carriers to be tested for ALT every three months during the first year to confirm that they are truly in the inactive carrier state, and then to repeat the test every 6-12 months [11]. More recently, the European Association for the Study of the Liver (EASL) clinical practice guideline recommends a minimal follow-up of one year, with serum ALT and HBV DNA levels being measured every three months to detect fluctuations of activity in patients with active HBeAg-negative CHB [12].

Serum ALT levels may remain within the normal range for long periods after reactivation of HBV replication [18]. In addition, the serum ALT level was found to be normal in 20%-30% of patients with HBeAg-negative CHB at presentation [13]. These features could result in some patients with HBeAg-negative CHB being misclassified as inactive carriers when only serum ALT levels (i.e., not HBV DNA levels) are monitored. Although these patients could be appropriately classified with serial monitoring of HBV DNA levels, this strategy might be impossible in some cases because HBV DNA assays are not widely available and they are expensive. In addition, it is still uncertain whether this expensive test is necessary for differentiating these two disease conditions. This study was therefore performed to establish a follow-up strategy for differentiating patients with HBeAg-negative CHB and true inactive carriers.

## Materials and Methods

### Patients

Between January 2006 and December 2007, consecutive untreated patients who were potential inactive carriers (defined as HBeAg-negativity, anti-HBe-positivity, normal ALT levels, and HBV DNA < 2000 IU/mL) were enrolled in this study. Exclusion criteria include previous diagnosis as liver cirrhosis or hepatocellular carcinoma and treatment with nucleoside analogues, interferon, or immunosuppressive agents; alcohol abuse (daily alcohol intake > 60 g); and positivity for anti-hepatitis C virus antibody or anti-HIV. Hepatitis B serologic markers including HBsAg, HBeAg, and anti-HBe were tested using commercially available enzyme-linked immunosorbent assay kits (Abbott Laboratories, North Chicago, IL, USA). This study was conducted in full conformance with the ethical guidelines ofthe1975 Helsinki Declaration and was approved by our Institutional Review Board. Written informed consent was obtained from each participant.

### Definition and classification

Patients were assessed at least every three months if there were any clinical indications for one year to determine if they were truly in the inactive carrier state. At each visit, serum ALT, bilirubin, albumin, and HBV DNA levels were checked. ALT, bilirubin, and albumin were measured by commercially available kits, with the upper limit of normal value (ULN) of ALT defined as 40 IU/L. HBV DNA quantification was performed by the COBAS TaqMan HBV DNA assay (Roche Diagnostics, Branchburg, NJ, USA; lower detection limit: 60 IU/mL). HBV reactivation was defined as elevation of the HBV DNA level to ≥ 2000 IU/mL. Patients were classified according to their serum ALT or HBV DNA level during the first year into the normal ALT group, when their ALT levels remained within normal range during the first year, or the abnormal ALT group, when their ALT levels increased to > 40 IU/L during the first year, and into true inactive carriers when their HBV DNA levels remained at < 2000 IU/mL during the first year or false inactive carriers when their HBV DNA levels increased to ≥2000 IU/mL during the first year. Patients were followed until HBV DNA reactivation developed.

### Statistical analysis

Continuous and categorical variables were expressed as Mean ± SD and number of patients (%), respectively. Differences between two groups were analyzed using the Mann-Whitney U or x(2) test. Area under the receiver operating characteristic curve (AUROC) analysis was performed to assess the predictive efficacy for HBV reactivation. Cutoff values of HBV DNA for predicting HBV reactivation within one year were chosen for maximizing the sum of sensitivity and specificity. Kaplan-Meier method was used to assess the cumulative incidence of HBV reactivation. Univariate and multivariate analyses were performed to identify the factors associated with the development of HBV reactivation or abnormal ALT levels. All statistical analyses were done by SPSS ver 13.0 (SPSS Inc., Chicago, IL, USA). A p value < 0.05 was considered statistically significant.

## Results

### Baseline characteristics

A total of 225 consecutive inactive carriers were enrolled in this study. Their Mean±SD age was 48.2 ± 12.6 years; 151 (67.1%) of studied patients were male. Seventeen (7.6%) patients were lost in follow-up in one year. HBV reactivation did not develop in these patients during a Mean ± SD follow-up of 138 ± 45 days. Although there was a trend for the Mean ± SD serum ALT level to be slightly lower in these patients than the remaining patients (18.6 ± 9.3 vs. 22.8 ± 8.7 IU/L, p = 0.058), the Mean ± SD serum HBV DNA level did not differ significantly between these two groups (449 ± 565 vs. 364 ± 483IU/mL; p = 0.314). These 17 patients were excluded from further analyses. The Mean ± SD age of the remaining 208 (140 male) patients who were followed for at least one year was 47.7 ± 12.6 years (Table 1). The Mean ± SD serum ALT and HBV DNA level was 22.8 ± 8.6 IU/L and 360 ± 482 IU/mL, respectively. The HBV DNA level was ≥ 200 IU/mL in 86 (41.3%) patients.

**Table 1:** Baseline characteristics of HBV carriers according to the development of abnormal ALT levels or HBV reactivation within one year

	**All patients **(No. = 208)	**Normal ALT a group **(No. = 190)	**Abnormal ALTa group **(No. = 18)	**p-value b**	**True inactive carriers **(No. = 167)	**False inactive carriers **(No. = 41)	**p-value c**
**Age **(years)	47.7 ± 12.6	48.1 ± 12.8	44.1 ± 9.7	0.114	48.0 ± 13.3	46.5 ± 9.6	0.242
**Male **[No. (%)]	140 (67.3)	127 (66.8)	13 (72.2)	0.642	114 (68.3)	26 (63.4)	0.553
**Diabetes **[No. (%)]	19 (9.2)	17 (9.0)	2 (11.1)	0.766	17 (10.2)	2 (5.0)	0.308
**BMI **(kg/m2)	23.6 ± 2.9	23.5 ± 2.9	24.9 ± 3.1	0.036	23.7 ± 3.1	23.4 ± 2.1	0.895
**ALT** (IU/L)	22.8 ± 8.6	22.0 ± 8.3	30.9 ± 7.8	< 0.001	22.2 ± 8.6	25.2 ± 8.3	0.031
**Bilirubin **(mg/dL)	0.8 ± 0.3	0.8 ± 0.3	0.8 ± 0.3	0.589	0.8 ± 0.3	0.8 ± 0.2	0.861
**Albumin **(g/dL)	4.6 ± 0.3	4.6 ± 0.3	4.6 ± 0.2	0.809	4.6 ± 0.3	4.6 ± 0.3	0.200
**Cholesterol **(mg/dL)	169.3 ± 30.4	168.5 ± 30.8	178.2 ± 25.0	0.184	169.0 ± 30.3	170.8 ± 31.2	0.982
**Triglyceride **(mg/dL)	99.9 ± 44.9	95.9 ± 39.7	141.7 ± 70.4	0.001	100.6 ± 45.7	96.8 ± 42.0	0.521
**Glucose **(mg/dL)	98.4 ± 21.4	98.6 ± 22.1	96.3 ± 11.9	0.900	99.9 ± 23.0	92.1 ± 11.1	0.005
**GGT **(IU/L)	32.9 ± 37.0	30.0 ± 32.6	63.2 ± 61.5	0.002	31.9 ± 37.7	37.1 ± 34.1	0.090
**HBVa DNA **(IU/mL)	360 ± 482	348 ± 476	485 ± 546	0.633	248 ± 385	813 ± 570	< 0.001

^a^ HBV: Hepatitis B virus; ALT: Alanine aminotransferase; BMI: Body mass index

^b^ Difference between normal ALT group and abnormal ALT group

^c^ Difference between true inactive carriers and false inactive carriers; normal ALT group: patients whose ALT level remained within normal range during the first year; abnormal ALT group: patients whose ALT level increased to > 40 IU/L during the first year; true inactive carriers: patients whose HBV DNA levels remained < 2000 IU/mL during the first year; false inactive carriers: patients whose HBV increased to ≥ 2000 IU/mL during the first year.

In univariate linear regression analysis, sex and body mass index (BMI) were found to be significantly correlated with serum ALT level, but not with age, presence of diabetes, and bilirubin, albumin, cholesterol, and HBV DNA levels. In multivariate linear regression analysis, BMI was the only independent factor significantly correlated with serum ALT level (β = 0.743; 95% confidence interval (CI): 0.347-1.138; p < 0.001) (Table 2).

**Table 2:** Univariate and multivariate linear regression analysis for baseline serum ALTa level

**Variable**	**p-value a**	**p-value b**	**β **(95% CI c)
**Age ** (years)	0.342		
**Sex**	0.007	0.083	2.206 ( 0.288 to 4.699)
**BMIc**(kg/m2)	< 0.001	< 0.001	0.743 (0.347 to 1.138)
**Diabetes**	0.027	0.114	
**Bilirubin **(mg/dL)	0.396		
**Albumin ** (g/dL)	0.735		
**Cholesterol **(mg/dL)	0.415		
**Triglyceride **(mg/dL)	0.024	0.323	
**HBV c DNA** (IU/mL)	0.404		

^a^ p values for univariate linear regression analysis

^b^ p values for multivariate linear regression analysis

^c^ ALT: Alanine aminotransferase; BMI: Body mass index; CI: Confidence interval; HBV: Hepatitis B virus

### Changes in serum ALT level within one year

Within one year, ALT level became abnormal in 18 (8.7%) patients (abnormal ALT group) but, remained within the ULN in 190 (91.3%) patients (normal ALT group). The prevalence of HBV reactivation within one year did not differ significantly (p = 0.129) between the abnormal ALT group (6 of 18 patients, 33%) and the normal ALT group (35 of 190 patients, 18%). At the time of abnormal ALT measurements, HBV DNA levels were > 2000 IU/L in only 6 of the 18 patients in the abnormal ALT group. BMI, baseline ALT and triglyceride (TG) levels differed significantly between the normal and abnormal ALT groups, while age, sex, diabetes status, and the baseline cholesterol, bilirubin, albumin, and HBV DNA levels did not (Table1). In multivariate regression analysis, the baseline ALT (β = 0.121; OR = 1.128; 95% CI: 1.055-1.027; p < 0.001) and TG (β = 0.015; OR = 1.015; 95% CI: 1.005-1.026; p = 0.003) levels were found independent predictive factors for an abnormal ALT.

### Development of HBV reactivation within one year

**Figure 1:**
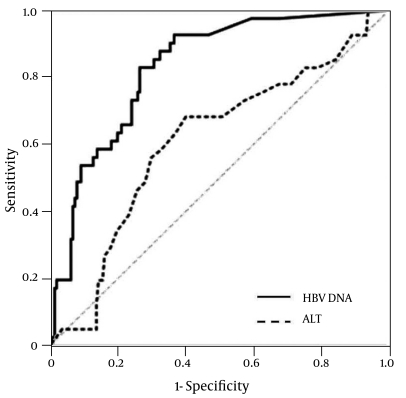
Area under receiver operating characteristic curve for hepatitis B virus (HBV) reactivation of baseline HBV DNA and ALT levels. ALT: Alanine aminotransferase

**Figure 2:**
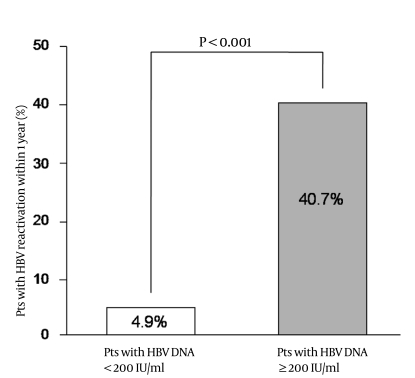
Incidence of hepatitis B virus (HBV) reactivation within one year according to the baseline HBV DNA level

HBV reactivation developed within one year in 41 (19.7%) patients (false inactive carriers): in 10, 14, 5, and 12 patients at 3, 6, 9, and 12 months, respectively. HBeAg remained negative after HBV reactivation in these patients. The serum ALT level was abnormal at the time of HBV reactivation in 6 (15%) of 41 patients who were false inactive carriers. The Mean ± SD baseline HBV DNA level (248 ± 385 IU/mL vs. 813 ± 570 IU/mL, p < 0.001) and ALT (22.2 ± 8.6 IU/L vs. 25.2 ± 8.3 IU/L, p = 0.031) levels differed significantly between true inactive carriers and false inactive carriers (Table1). The area under the receiver operator characteristic (AUROC) of the baseline HBV DNA level and for predicting HBV reactivation during the first year was 0.831 (95% CI: 0.767-0.895) (Figure. 1). With a cutoff value of 200 IU/mL, the sensitivity, specificity, positive predictive value (PPV), and negative predictive value (NPV) were 85.4%, 69.5%, 40.7%, and 95.1%, respectively (Table3). HBV reactivation developed in six (4.9%) patients with a baseline HBV DNA level of < 200 IU/mL, and in 35 (41%) of patients with a baseline HBV DNA level of ≥ 200 IU/mL (p < 0.001) (Figure. 2).

**Table 3:** Sensitivity, specificity, positive and negative predictive values for predicting HBVa reactivation within one year according to the different cutoff values of serum HBV DNA level

**Cutoff value** (IU/mL)	**Sensitivity** (%)	**Specificity** (%)	**PPV a** (%)	**NPV a** (%)
20	97.6	32.9	26.3	98.2
50	97.6	38.9	28.2	98.5
100	92.7	59.3	35.8	97.1
200	85.4	69.5	40.7	95.1
500	61.0	81.4	44.6	89.5
1,000	41.5	93.4	60.7	86.7

^a^ HBV: HepatitisB virus, PPV: Positive predictive value, NPV: Negative predictive value

The AUROC of the serum ALT level for predicting HBV reactivation during the first year was 0.609 (95% CI: 0.512-0.706) (Figure. 1). With the optimal cutoff value of 22.5 IU/L, the sensitivity, specificity, positive predictive value, and negative predictive value were 68.3%, 59.9%, 29.5%, and 88.5%, respectively.

### Follow -Up

**Figure 3:**
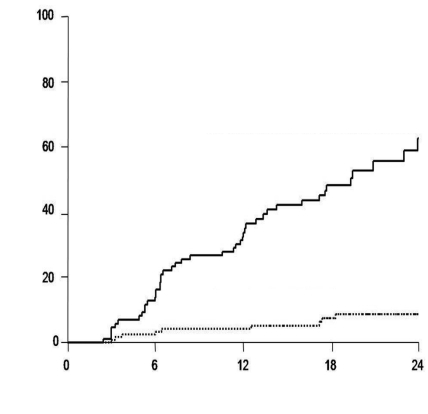
Cumulative incidence of hepatitis B virus (HBV) reactivation in inactive HBV group according to the baseline HBV DNA level

**Figure 4:**
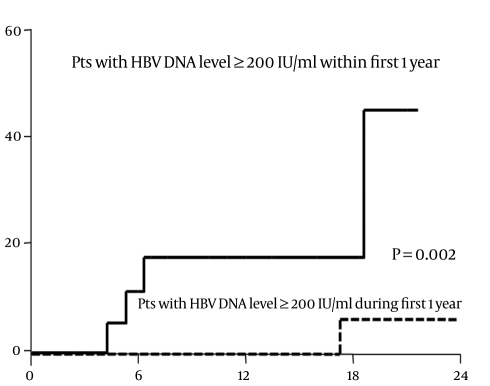
Cumulative incidence of hepatitis B virus (HBV) reactivation in patients whose HBV DNA levels remained < 200 IU/mL during the first year and in those whose HBV DNA levels increased to ≥ 200 IU/mL during the first year

The Mean ± SD total follow-up was 544 ± 243 days in all enrolled patients; it was 622 ± 199 days in true inactive carriers. HBsAg seroconversion was not observed in all enrolled patients during the follow-up; HBV reactivation developed in 55 patients. The cumulative incidences of HBV reactivation in potential inactive carriers were 8.7%, 16.3%, 24.4%, and 30.5% at 6, 12, 18, and 24 months, respectively. The cumulative incidence of HBV reactivation was significantly (p < 0.001) higher in patients with a baseline HBV DNA level of ≥ 200 IU/mL (33.7% and 62.6% at 12 and 24 months, respectively) than in those with a baseline HBV DNA level of < 200 IU/mL (4.1% and 8.7% at 12 and 24 months, respectively) (Figure 3). Among 55 patients with HBV reactivation, serum ALT level increased in 17 patients during a Mean ± SD follow-up of 525 ± 349 days after HBV reactivation. The Mean ± SD serum ALT and HBV DNA levels were 109 ± 69 IU/L and 5.7 ± 1.2 log10 IU/mL, respectively, at the time of ALT elevation. Ninety-five patients whose baseline HBV DNA level was < 200 IU/mL in the inactive HBV group were followed for a Mean ± SD of 331 ± 186 more days after the first year. The HBV DNA level remained at < 200 IU/L during the first year in 75 patients and increased to ≥ 200 IU/mL in 20 patients. During the follow-up, HBV reactivation developed in 0%, 11.5%, 17.8%, and 17.8% at 3, 6, 9, and 12 months after the first year in 20 patients whose HBV DNA levels increased to ≥ 200 IU/mL during the first year; it did not develop in 75 patients whose HBV DNA levels remained < 200 IU/mL during the first year (p = 0.002) (Figure 4).

## Discussion

The current aim of managing patients with chronic HBV infection is to prevent the disease progression into cirrhosis and HCC, and to decrease other liver-related mortality by sustained suppression of HBV replication [11][12]. Several recent studies have indicated that the HBV DNA level is directly correlated with the risk of disease progression in HBV carriers. Recent large prospective cohort studies have suggested that the HBV DNA level is a prominent risk for developing HCC [17] and liver cirrhosis [16] independently of the HBeAg status and serum ALT level. Significant dose-response relationships between the serum HBV DNA level and the risk of HCC [17] and liver cirrhosis [16], have been found for HBV DNA levels above 2000 IU/mL [16][17]. The cutoff value for the HBV DNA level of an inactive carrier has recently been recommended to be 2000 IU/mL. This management target could be considered in the long-term maintenance of patients with chronic HBV infection in the inactive carrier state. The prognosis of inactive carriers is generally favorable. A long-term follow-up study of HBsAg-positive blood donors suggested that the survival rate did not differ between HBsAg-positive blood donors and HBV-uninfected controls over a 30-year period [10]. In addition, most patients who had experienced spontaneous HBeAg seroconversion exhibited sustained remission during a median follow-up of nine years [9]. However, some inactive carriers experience reactivation of HBV replication, after which the prognosis usually worsens [9][19]. Therefore, predicting the probability of HBV reactivation in HBV carriers with an HBV DNA level < 2000 IU/mL is very important when attempting to design an appropriate follow-up schedule and to estimate prognosis.

The AASLD guideline recommends that inactive carriers should be monitored (including ALT levels) every three months during the first year to confirm that they are truly in the "inactive carrier state" and every 6-12 months thereafter. It is recommended that HBV DNA levels be monitored if the elevation of serum ALT level persists or recurs [11]. In the present study, HBV reactivation developed in 19.7% of those in the normal ALT group, who could be considered to be truly in the "inactive carrier state" according to the AASLD guideline, while the HBV DNA level remained < 2000 IU/mL during the first year in 33.3% of those in the abnormal ALT group. In addition, the serum ALT level was not useful for detecting or predicting HBV reactivation in our study. The AUROC for HBV reactivation within one year was only 0.609. HBV DNA levels remained stable in 66.7% of patients with abnormal ALT levels, and HBV reactivation developed in 18.4% of patients with an ALT level that remained normal for one year. Using the recently recommended ULNs of ALT (30 IU/L for men and 19 IU/L for women) did not improve the sensitivity, specificity, PPV, and NPV, which were 41.5%, 83.6%, 27.4%, and 83.6%, respectively (data not shown). Our results are consistent with a recent study finding that one-third of patients with HBeAg-negative CHB experienced serum HBV DNA levels < 2000 IU/mL at least once during their follow-up, and that 22% of these cases presented with persistently abnormal ALT levels [8]. Other studies have also suggested that a normal ALT level alone is not an accurate indicator of inactive disease [20][21]. Therefore, only serial measurements of serum ALT level might not be enough for differentiating HBV carriers who are "truly" in the inactive HBV replication state from HBeAg-negative CHB patients. On the other hand, the EASL guideline suggests that careful assessment of inactive carriers is needed, with a minimal follow-up of one year and serum ALT and HBV DNA levels being measured every three months usually allowing fluctuations of activity in patients with active HBeAg-negative CHB to be detected [12]. However, this strategy with frequent monitoring using expensive HBV DNA tests might not be cost-effective, and in some cases might even be impossible for the high cost. Therefore, a more efficient follow-up strategy is needed.

In the present study, baseline HBV DNA and ALT levels differed significantly between true inactive and false inactive carriers. However, the predictive efficacy of the baseline ALT level for false inactive carriers was low, with an AUROC of only 0.609. In contrast, the baseline HBV DNA level was found to be very useful for discriminating false inactive carriers from true inactive carriers-most of inactive carriers could be discriminated using a cutoff value of 200 IU/mL. In addition, HBV reactivation did not develop during the following year in HBV carriers whose HBV DNA level remained < 200 IU/mL within the first year. However, the risk of HBV reactivation increased when the HBV DNA level was ≥ 200 IU/mL at any time during follow-up. Therefore, the follow-up strategy for detecting inactive carriers might need to differ based on the HBV DNA levels: in HBV carriers with an HBV DNA level < 200 IU/mL, yearly follow-up of HBV DNA levels seems to be sufficient for detecting HBV reactivation; if HBV DNA level is ≥ 200 IU/mL, 6-monthly follow-up of HBV DNA levels might be more appropriate.

A recent study suggested that the serum HBV DNA level was higher in those with high-normal ALT (≥ 0.5×ULN) than in those with low-normal ALT (< 0.5×ULN) [5], which is inconsistent with the results of our study (where the serum ALT level was not correlated with the HBV DNA level). This discrepancy might be due to the use of different definitions for inactive carriers. The range of HBV DNA levels was narrower in the present study because we defined the ULN of the HBV DNA level in an inactive carrier as < 2000 IU/mL, while the previous study enrolled patients with HBeAg-negativity and persistently normal ALT level regardless of their HBV DNA level [5]. This discrepancy in the study design might have lessened the effect of HBV DNA level on serum ALT level in our patients. However, consistent finding of the previous study was that a serum HBV DNA level ≥ 2000 IU/mL was independently associated with high-normal ALT levels [5]. Our study excluded patients with HBV DNA level ≥ 2000 IU/mL. Another possible explanation for the observed discrepancy is that in our study the baseline ALT and TG levels were significant predictive factors for an abnormal ALT groups and only BMI being significantly associated with the baseline ALT level. Elevated TG level and BMI are associated with hepatic steatosis in CHB patients [22][23][24]. Because BMI was not reported for the previous studies, direct comparison was not possible. However, according to our results, the effect of HBV DNA level was minimal and serum ALT levels were influenced mainly by fatty liver disease in HBV carriers with an HBV DNA level < 2000 IU/mL. That is, serum ALT level is influenced by not only the HBV DNA level but also the degree of fatty liver disease, and this might have also led to the low predictive value of serum ALT levels for HBV reactivation in our results. The main limitation of our study was the short duration of follow-up in our patients: all enrolled patients and true inactive carriers were followed for a Mean ± SD of 544 ± 243 and 622 ± 199 days, respectively. Further studies with longer follow-up duration are needed to confirm our results.

In conclusion, we found that HBV DNA level is a useful mean for discriminating patients with HBeAg-negative CHB from true inactive carriers. The follow-up strategy applied to inactive carriers need to vary according to their HBV DNA levels.
